# 供者CD7 CAR-T细胞治疗异基因造血干细胞移植后急性T淋巴细胞白血病复发1例

**DOI:** 10.3760/cma.j.issn.0253-2727.2023.01.016

**Published:** 2023-01

**Authors:** 慧娟 黄, 丹 刘, 源兵 吴, 珊珊 姜, 雅雪 吴, 梦佳 侯, 晓慧 胡, 骁 马

**Affiliations:** 1 苏州大学附属第一医院，苏州 215006 The First Affiliated Hospital of Soochow University, Suzhou 215006, China; 2 苏州弘慈血液病医院，苏州 215006 Soochow Hopes Hematology Hospital, Suzhou 215006, China

患者，男性，28岁，2019年诊断为急性T淋巴细胞白血病（T-ALL），应用Hyper-CVAD方案治疗，A、B方案交替进行各2个疗程后，获持续分子学缓解。2019年10月予改良BU/CY方案预处理后，行异基因造血干细胞移植，供者为其胞弟，干细胞来源为骨髓+外周血。予吗替麦考酚酯、甲泼尼龙、环孢素A预防急性移植物抗宿主病（aGVHD），移植后1个月出现皮肤及肝脏GVHD，经治疗后好转。长期口服甲泼尼龙、环孢素A控制GVHD并门诊随访减量，后调整为甲泼尼龙及他克莫司较小剂量维持，期间皮肤GVHD反复，皮肤慢性GVHD持续。

2021年2月（移植后15个月）时患者出现本病复发。查体：左侧肘部及左胫骨前缘皮下肿块，拳头大小；躯干及四肢大量斑片状充血性皮疹。外周血涂片：原始细胞5％。骨髓象：原始幼稚淋巴细胞占0.255，提示T-ALL复发。流式细胞术：分析16.73％的幼稚细胞群，抗原表达：cCD3^+^、CD34^±^、CD13^±^、CD11b^±^、CD38^+^、CD7^+^、CD5^±^、CD2^−^、CD3^−^、CD4^−^、CD8^−^，为T系抗原表达。TCR重排阳性，WT1 5.99％，供者细胞嵌合率（STR）（骨髓）67.2％。MRI：左侧肘关节及左胫骨上段周围，左侧腘窝多发软组织占位，左侧肱骨、尺骨、桡骨、左侧胫腓骨、股骨及髌骨多发骨质破坏，符合病理性白血病表现。考虑患者髓内复发、髓外浸润表现。停抗皮肤GVHD治疗。先后予地西他滨+AAG、大剂量甲氨蝶呤、IVP+阿糖胞苷方案治疗，左肘及左侧胫前肿块较前明显缩小。治疗结束后4 d出现粒细胞缺乏（粒缺）伴发热，并发感染性休克，积极抗感染后好转。治疗结束后11 d复查：骨髓形态缓解中；微小残留病（MRD）1.4×10^−4^；STR（骨髓）：96.1％，左肘及左侧胫前肿块较前稍有增大。治疗结束后21 d CT示：左侧胫前肿块大小约为125 mm×76 mm×47 mm，左肘部肿块98 mm×74 mm×48 mm，考虑髓外浸润进展。予信迪利单抗100 mg，第1、10天各1剂，患者出现1级发热、2级皮肤和胃肠道免疫相关不良反应，予以甲泼尼龙+芦可替尼干预后好转。予FC方案预处理。复查CT示：左侧胫前肿块125 mm×61 mm×36 mm（较前缩小38％），左肘部肿块90 mm×61 mm×49 mm（较前缩小23％），根据淋巴瘤Lugano疗效CT评价标准评估病情稳定。MRD<2.3×10^−4^；WT1 0.17％；TCR重排阴性。STR（外周血）：98.9％。回输供者CD7 CAR-T细胞，细胞活率89.09％，转导效率13.22％；总量1×10^7^/kg，按照10％～30％～60％分3 d回输。回输结束后第2天双下肢新发皮疹，无水泡及皮肤破溃，面积<20％，为Ⅰ级皮肤GVHD，间断予地塞米松5 mg；患者出现粒缺伴发热，根据咽拭子培养及药敏调整抗感染方案后仍有间断发热。回输结束后5 d全身散在皮疹，双下肢显著伴水疱，皮疹水疱液IL-6 22 000 ng/L（参考范围<20.0 ng/L），GVHD组套：游离肿瘤形成抑制素2（sST2）46 µg/L，考虑皮肤GVHD加重。发热>38 °C，胸闷（低流量3 L/min吸氧），血IL-6 300 ng/L，考虑2级细胞因子释放综合征（CRS）。加用他克莫司1.5 mg/d（血药浓度维持4～6 µg/L），芦可替尼 5 mg每日2次，皮疹处予对症处理，后皮疹未进行性加重，血象逐步回升。间断反复予小剂量激素治疗，CRS持续5 d后IL-6恢复正常。CAR-T细胞治疗14 d复查示骨髓缓解，左侧胫前肿块50 mm×32 mm×20 mm，左肘部肿块32 mm×20 mm×15 mm。髓外包块缩小90％以上，评估达部分缓解（PR）。

讨论：本研究提示PD-1单抗联合CD7 CAR-T对难治性/复发T-ALL患者有一定疗效，可作为一种挽救性治疗策略，芦可替尼可协助控制治疗相关不良反应，提高治疗安全性。

